# Using Laboratory Test Results for Surveillance During a New Outbreak of Acute Hepatitis in 3-Week- to 5-Year-Old Children in the United Kingdom, the Netherlands, Ireland, and Curaçao: Observational Cohort Study

**DOI:** 10.2196/55376

**Published:** 2024-12-23

**Authors:** Maaike C Swets, Steven R Kerr, Brian MacKenna, Louis Fisher, Merel van Wijnen, Diederik Brandwagt, Paul W Schenk, Pieter Fraaij, Leonardus G Visser, Sebastian Bacon, Amir Mehrkar, Alistair Nichol, Patrick Twomey, Philippa C Matthews, ISARIC4C Hepatitis Study Group, Iain Jones, Malcolm G Semple, Geert H Groeneveld, Ben Goldacre, Iain Jones, J Kenneth Baillie

**Affiliations:** 1 Department of Infectious Diseases, Leiden University Medical Center, Leiden University, Albinusdreef 2, Leiden, Netherlands; 2 Roslin Institute, University of Edinburgh, Edinburgh, United Kingdom; 3 Bennett Institute for Applied Data Science, Nuffield Department of Primary Care Health Sciences, University of Oxford, Oxford, United Kingdom; 4 Department of Clinical Chemistry, Meander Medical Centre, Amersfoort, Netherlands; 5 Centre for Infectious Disease Control, National Institute for Public Health and the Environment (RIVM), Bilthoven, Netherlands; 6 Department of Clinical Chemistry and Laboratory Medicine, Leiden University Medical Center, Leiden University, Leiden, Netherlands; 7 Department of Pediatrics, Erasmus Medical Center, Rotterdam, Netherlands; 8 Department of Viroscience, Erasmus Medical Center, Rotterdam, Netherlands; 9 University College Dublin-Clinical Research Centre, St Vincent’s University Hospital, Dublin, Ireland; 10 The Australian and New Zealand Intensive Care Research Centre, Monash University, Melbourne, Australia; 11 The Francis Crick Institute, London, United Kingdom; 12 see Acknowledgments; 13 Institute of Infection, Veterinary and Ecological Sciences, Faculty of Health and Life Sciences, University of Liverpool, Liverpool, United Kingdom; 14 Biochemistry Laboratory, Queen Elizabeth University Hospital, Glasgow, United Kingdom; 15 Baillie Gifford Pandemic Science Hub, Centre for Inflammation Research, University of Edinburgh, Edinburgh, United Kingdom; 16 Intensive Care Unit, Royal Infirmary of Edinburgh, Edinburgh, United Kingdom; 17 Medical Research Council (MRC) Human Genetics Unit, Institute for Genetics and Molecular Medicine, University of Edinburgh, Edinburgh, United Kingdom

**Keywords:** pediatric hepatitis, disease surveillance, outbreak detection, pandemic preparedness, acute hepatitis, children, data analytics, hospital, laboratory, all age groups, pre-pandemic, United Kingdom, Netherlands, Ireland Curacao, single center, federated analytics, pandemic surveillance, outbreaks, public health

## Abstract

**Background:**

In March 2022, a concerning rise in cases of unexplained pediatric hepatitis was reported in multiple countries. Cases were defined as acute hepatitis with serum transaminases >500 U/L (aspartate transaminase [AST] or alanine transaminase [ALT]) in children aged 16 years or younger. We explored a simple federated data analytics method to search for evidence of unreported cases using routinely held data. We conducted a pragmatic survey to analyze changes in the proportion of hospitalized children with elevated AST or ALT over time. In addition, we studied the feasibility of using routinely collected clinical laboratory results to detect or follow-up the outbreak of an infectious disease.

**Objective:**

We explored a simple federated data analytics method to search for evidence of unreported cases using routinely held data.

**Methods:**

We provided hospitals with a simple computational tool to enable laboratories to share nondisclosive summary-level data. Summary statistics for AST and ALT measurements were collected from the last 10 years across all age groups. Measurements were considered elevated if ALT or AST was >200 U/L. The rate of elevated AST or ALT test for 3-week- to 5-year-olds was compared between a period of interest in which cases of hepatitis were reported (December 1, 2021, to August 31, 2022) and a prepandemic baseline period (January 1, 2012, to December 31, 2019). We calculated a *z* score, which measures the extent to which the rate for elevated ALT or AST was higher or lower in the period of interest compared to a baseline period, for the 3-week- to 5-year-olds.

**Results:**

Our approach of sharing a simple software tool for local use enabled rapid, federated data analysis. A total of 34 hospitals in the United Kingdom, the Netherlands, Ireland, and Curaçao were asked to contribute summary data, and 30 (88%) submitted their data. For all locations combined, the rate of elevated AST or ALT measurements in the period of interest was not elevated (*z* score=−0.46; *P=*.64). Results from individual regions were discordant, with a higher rate of elevated AST or ALT values in the Netherlands (*z* score=4.48; *P*<.001), driven by results from a single center in Utrecht. We did not observe any clear indication of changes in primary care activity or test results in the same period.

**Conclusions:**

Hospital laboratories collect large amounts of data on a daily basis that can potentially be of use for disease surveillance, but these are currently not optimally used. Federated analytics using nondisclosive, summary-level laboratory data sharing was successful, safe, and efficient. The approach holds potential as a tool for pandemic surveillance in future outbreaks. Our findings do not indicate the presence of a broader outbreak of mild hepatitis cases among young children, although there was an increase in elevated AST or ALT values locally in the Netherlands.

## Introduction

In March 2022, a series of cases of severe hepatitis in young children was recognized in Central Scotland [1]. As of August 26, 2022, a total of 1115 probable cases were reported—acute hepatitis (not caused by hepatitis A-E virus) with serum transaminase >500 U/L (aspartate transaminase [AST] or alanine transaminase [ALT]) in children aged 16 years or younger [2]. In this period, 22 children died in 35 countries [1-3] and 47 (4%) have undergone emergency liver transplants [3]. The majority of children meeting the case definition were younger than 5 years (range 1 month to 16 years) [1]. No new cases were reported since the late summer of 2022 [4]. We have recently reported evidence that the outbreak was caused by adeno-associated virus 2 (AAV2), in some cases in combination with adenovirus (HAdV) and human herpesvirus 6B [5-7]. AAV2 relies on coinfection with a helper virus for replication, such as HAdV or herpes viruses [6].

In the majority of outbreaks of infectious diseases, the number of mild cases is much higher than the number of severe cases [8]. In this hepatitis outbreak, milder, self-limiting cases (eg, without severe symptoms or jaundice) have not been reported. However, it is possible that this outbreak has been more widespread than recorded data suggest. Assessing the magnitude of the outbreak is crucial for understanding pathophysiological mechanisms and considering whether there is a future need to identify mild cases in the early stages to prevent clinical deterioration.

Hepatitis refers to inflammation of the liver and is characterized by an increase in transaminase levels, for which quantitative recordings are available in clinical laboratory databases [9,10]. These routinely collected laboratory data are rarely exploited for public health surveillance, but could be used as an opportunistic measure for disease surveillance in new or periodic outbreaks. In order to be effective, the biomarkers should have a clear relationship with the disease of interest and this relationship should be specific. For example, C-reactive protein can be increased as a consequence of many different disease processes, and is, therefore, less suitable for disease surveillance [11]. Routinely collected laboratory data could also be used in widespread poisoning incidents (eg, acute kidney injury linked to the consumption of cough syrup medication in Indonesia and Gambia in 2022) [12,13]. To our knowledge, this is the first study using routinely collected laboratory data to detect and follow-up on the hepatitis outbreak in children.

In order to assess any potential rise in the number of mild hepatitis cases, as measured by elevated serum transaminases, we conducted a pragmatic survey to analyze changes in the proportion of hospitalized children with elevated AST or ALT over time. Even though there are many different causes for an increase in ALT or AST, the proportion of elevated measurements relative to all measurements is expected to be stable over extended periods, especially in young children, where the threshold to take blood samples is relatively high. In addition, we studied the feasibility of using routinely collected clinical laboratory results to detect or follow-up the outbreak of an infectious disease. Locally aggregating patient-level data to anonymous summary data that can be easily shared between hospitals could be a simple and quick method to assist with disease surveillance.

## Methods

### Overview

We pooled nondisclosive summary data from 29 hospital clinical chemistry laboratory databases from the United Kingdom (including data from England, Scotland, and Wales), the Netherlands, and Ireland. In a sensitivity analysis, we also included data from a 30th hospital, in Curaçao.

### Data Collection

Instructions for data collection and a script to summarize the raw data were posted on the isaric4c website. Hospitals were contacted and asked to contribute data through different methods (eg, personal network, ISARIC4C network, social media, ISARIC4C website, and professional associations). Participating centers were asked to run the script on local datasets containing all AST and ALT measurements for all hospitalized patients from January 2012 until the day of data extraction (March-December 2022), as well as the sample date and date of birth or age. Data collection can be done automatically in most laboratory systems. The script created completely anonymous summary data consisting of counts, means, and variances in the four categories of (1) AST measurements, (2) first elevated AST measurements, (3) ALT measurements, and (4) first elevated ALT measurements

Measurements were considered elevated if ALT or AST was >200 U/L. While there is variation by region, the upper limit of normal (ULN) for AST and ALT in children is usually around 50 U/L and 40 U/L, respectively, meaning that our cutoff is roughly 4‐5 times the ULN, and is typically considered a mild to moderate elevation [14]. These statistics were calculated for each month in our study period in the age groups of younger than 3 weeks old, 3 weeks to 5 years old, 6‐16 years old, 17‐50 years old, and older than 50 years. The 3-week- to 5-year-old age group was of primary interest. Any measurements for an individual who had previously had an elevated measurement of ALT or AST during the study period were removed. In Wales (United Kingdom), pooled data were collected for 4 different health boards. Details on the different health boards can be found in the supplement.

### Cases of Non–A-E Hepatitis

The monthly incidence of children meeting the case definition for non–A-E hepatitis was also collected. If there was a larger outbreak of mild cases of hepatitis, this most likely occurred in the same time period in which the cases of children presenting with non–A-E hepatitis were reported. For the Netherlands, these data were provided by the National Institute for Public Health and the Environment (RIVM) and the Dutch Society of Pediatrics. For other countries, these data were retrieved from several published studies [4,15].

### Primary Care Population Data

In parallel, with the approval of National Health Service (NHS) England, we used the OpenSAFELY secure health analytics platform to conduct a retrospective cohort study of liver function tests across the full pseudonymized patient primary care records held by the electronic health record provider TPP, covering 40% of general practices in England. Using data for the period between April 1, 2017, and March 31, 2022, we identified individuals aged 30 years or younger registered at a general practitioner at the beginning of each month. In these patients, we then identified anyone with any of the following liver function tests: ALT, AST, and bilirubin. For AST and ALT tests, we identified the number of tests in the clearly pathological range (>500 U/L), as set out in the World Health Organization (WHO) working case definition [2]. For bilirubin tests, the number of tests out of range was calculated using the upper reference range attached to the laboratory result.

### Statistical Analysis

Summary data from each center were combined to calculate pooled counts, means, and variances. We tabulated the number of individuals with AST and ALT measurements by location and plotted the number of tests per month. We plotted rates for elevated AST and ALT measurements by month in each age group as the proportion of the total number of tests. For example, a rate of 0.05 means that 5% of all tests were elevated. We also plotted the mean of AST and ALT among all those who had elevated measurements by month in each age group. This analysis was carried out for each center individually and for various pooled datasets (eg, all locations in England, Scotland, and Wales were pooled for the UK analysis). We calculated a *z* score, which measures the extent to which the rate for elevated ALT or AST was higher or lower in the period of interest compared to a baseline period, for the 3-week- to 5-year-olds. Since health-seeking behavior might have changed during the COVID-19 pandemic, a pre–COVID-19 period was used as a baseline period (January 1, 2012, to December 31, 2019). The period of interest included months in which children meeting the case definition were recorded (December 1, 2021, to August 31, 2022). For example, if the *z* score was 0.4 for a certain location for ALT, the rate for elevated ALT was 0.4 SDs higher in the period of interest compared to the baseline period.

### Ethical Considerations

For the analysis of summary hospital data, since only completely anonymous summary data were collected and shared, no ethics approval was needed. Given that only completely anonymous summary data were collected, the need for informed consent was waived. The openSAFELY study was approved by the Health Research Authority (REC reference 20/LO/0651). Given that only anonymous summary data of routine clinical results were used, there was no informed consent requirement.

## Results

### Overview

Between May 2022 and July 2022, 30 (88%) of 34 contacted hospitals contributed summary data. After data collection, preparing and aggregating the data using the script provided on the website typically took less than an hour, based on received feedback.

To conduct the analysis, we selected July 2022 as the cutoff date because the majority of hospitals did not have data available beyond that time. The number of AST and ALT tests performed in 3-week- to 5-year-olds at each site can be found in Table 1. The change in the number of tests over time in 3-week- to 5-year-olds in the United Kingdom, the Netherlands, and overall can be seen in Figure S1 in Multimedia Appendix 1. In the United Kingdom, there is a gradual increase in the number of ALT tests over time, with a significant drop at the start of the COVID-19 pandemic early in 2020 (Figure S1A in Multimedia Appendix 1). The sharp increase in 2018 is caused by data availability—both Edinburgh and the Highlands provided data from 2018 onward. The number of tests in the Netherlands is relatively stable over time, with an increase in both AST and ALT in 2018 (Figure S1B in Multimedia Appendix 1). When combining data for all available locations, there was an increase in the number of tests for both AST and ALT in 2014, because only data from the Netherlands were available for 2012‐2014. The number of AST tests has been relatively stable after 2014, while there has been a strong increase in ALT tests over time, mostly caused by the increase in tests in the United Kingdom (Figure S1C in Multimedia Appendix 1).

**Table 1.** Counts of elevated (>200 U/L) AST^a^ and ALT^b^ tested and total number of tests for the 3-week- to 5-year-olds (some hospitals did not admit any children in the 3-week- to 5-year-olds).

Location	Elevated AST, n	Total AST, n	Elevated ALT, n	Total ALT, n
Banbury, United Kingdom	0	0	0	0
Edinburgh, United Kingdom	86	349	900	19,255
Glasgow, United Kingdom	829	27,337	640	28,293
Highlands, United Kingdom	2	20	20	1778
Oxford, United Kingdom	6	15	39	2230
ABU^c^, ^d^United Kingdom	14	113	61	4333
CVU^e^, ^d^United Kingdom	31	272	224	8594
CTM^f^, ^d^United Kingdom	8	94	35	4411
SBU^g^, ^d^United Kingdom	9	74	45	4138
Amersfoort, the Netherlands	13	364	10	412
Amsterdam (1), the Netherlands	193	2827	196	3734
Amsterdam (2), the Netherlands	90	2911	82	3489
Groningen, the Netherlands	378	3020	233	3020
Den Bosch, the Netherlands	4	266	11	304
The Hague, the Netherlands	23	649	21	736
Leiden, the Netherlands	150	2942	73	3063
Nijmegen, the Netherlands	3	386	3	537
Utrecht, the Netherlands	604	19,711	621	21,606
Dublin, Ireland	0	0	0	1
Willemstad, Curaçao	9	217	5	216

a AST: aspartate transaminase.

b ALT: alanine transaminase.

c ABU: Aneurin Bevan University Health Board.

d Pooled data from health boards.

e CVU: Cardiff and Vale University Health Board.

f CTM: Cwm Taf Morgannwg University Health Board.

g SBU: Swansea Bay University Health Board.

Since ALT was measured more frequently than AST (see Table 1), the results for AST are presented in Multimedia Appendix 1. Data from Ireland are presented in the results with all data combined, as not enough data were available to present results on a country level. A hospital in Willemstad, Curaçao also provided us with data, but because only a few years of data were available, there were no recorded cases of hepatitis and Curaçao is a geographical outlier compared to the other contributing locations, we present the results including the data from Curaçao in a sensitivity analysis that can be found in Multimedia Appendix 1.

### ALT

**Figure 1.**
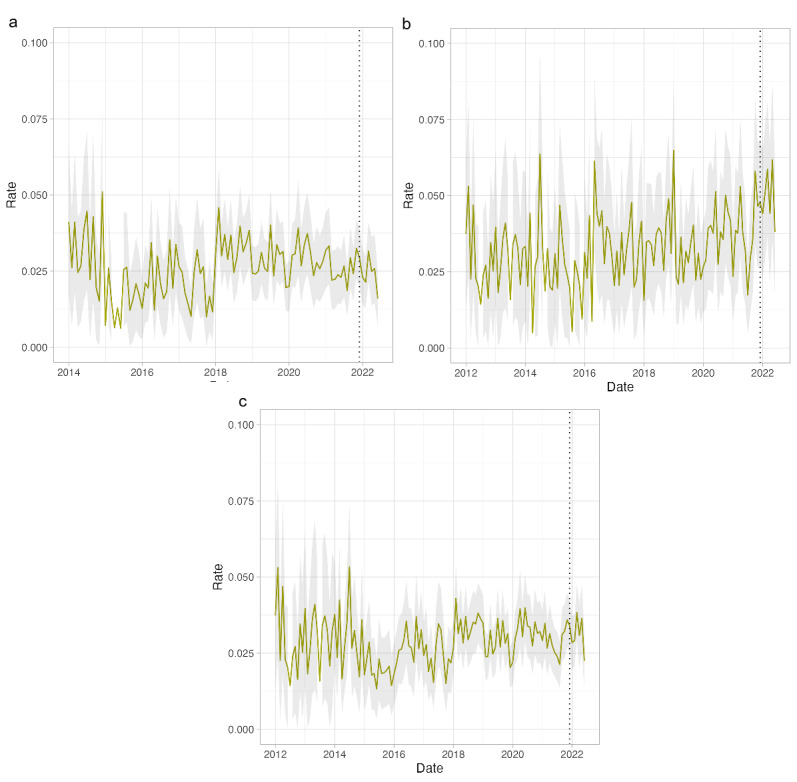
Rates for elevated (>200 U/L) alanine transaminase levels in the 3-week- to 5-year-old age group in the United Kingdom, the Netherlands, and all. Shaded area represents the 95% CI. The dotted line represents the first month in which cases of non–A-E hepatitis were reported.

First, we examined rates for elevated ALT values for 3-week- to 5-year-olds by location (Figure 1). There is a possible trend toward an increase in the rate for elevated ALT in our period of interest in the Netherlands, at the end of 2021 or the start of 2022 (Figure 1B), but this is not seen in the United Kingdom (Figure 1A). For the United Kingdom, the rate increased at the start of 2018 (Figure 1A). In the Netherlands, a similar increase takes place at the start of 2016 (Figure 1B). When pooling all available data, the increased rate is seen from 2018 onward (Figure 1C). Results for all age groups can be found in Figure S3 in Multimedia Appendix 1.

**Figure 2.**
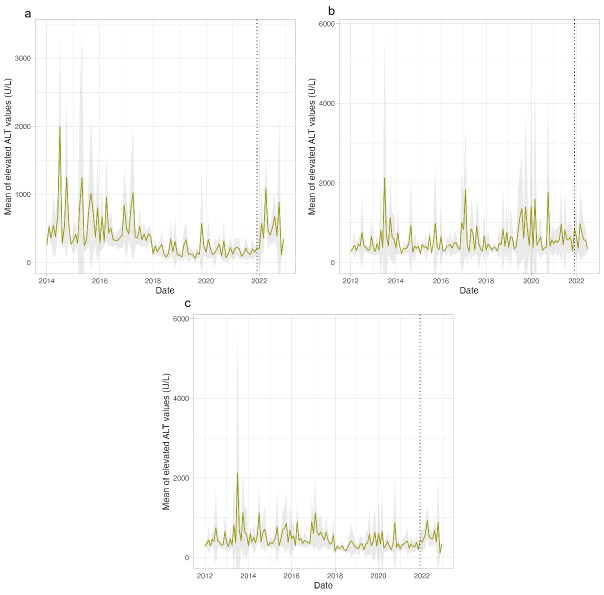
Mean ALT value (U/L) in the 3-week- to 5-year-olds with elevated (>200 U/L) ALT measurements in the United Kingdom, the Netherlands, and all. Shaded area represents the 95% CI. The dotted line represents the first month in which cases of non–A-E hepatitis were reported. ALT: alanine transaminase.

In 3-week- to 5-year-olds with an elevated ALT measurement, the mean ALT values were compared over time in the different contributing locations (Figure 2). A clear increase at the start of 2022 is seen in the United Kingdom (Figure 2A), but this increase is similar to or smaller than earlier peaks between 2014 and 2018. The same trend can be seen in the Netherlands, with an increase at the start of 2022, but similar increases happened in the earlier years (Figure 2B). Finally, when pooling all available data, an increase in the mean ALT value can be seen at the start of 2022, again with similar or higher peaks in earlier years (Figure 2C).

**Figure 3.**
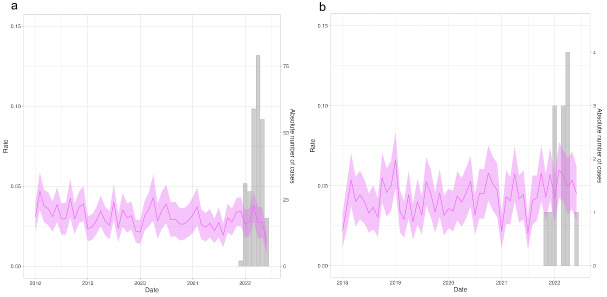
Overlap between rate of elevated (>200 U/L) ALT or AST levels from 2018 onward in 3-week- to 5-year-olds (left y-axis, line, and 95% CI) and the absolute number of cases presenting with non–A-E hepatitis in the United Kingdom and the Netherlands (right y-axis, bars). ALT: alanine transaminase; AST: aspartate transaminase.

The overlap between rates for elevated transaminases (elevated ALT, AST, or both) in children aged 3 weeks to 5 years and cases of (non–A-E) hepatitis can be seen in Figure 3. In both the United Kingdom and the Netherlands, there was no increase in the rate of elevated ALT or AST in the period in which cases were reported.

**Figure 4.**
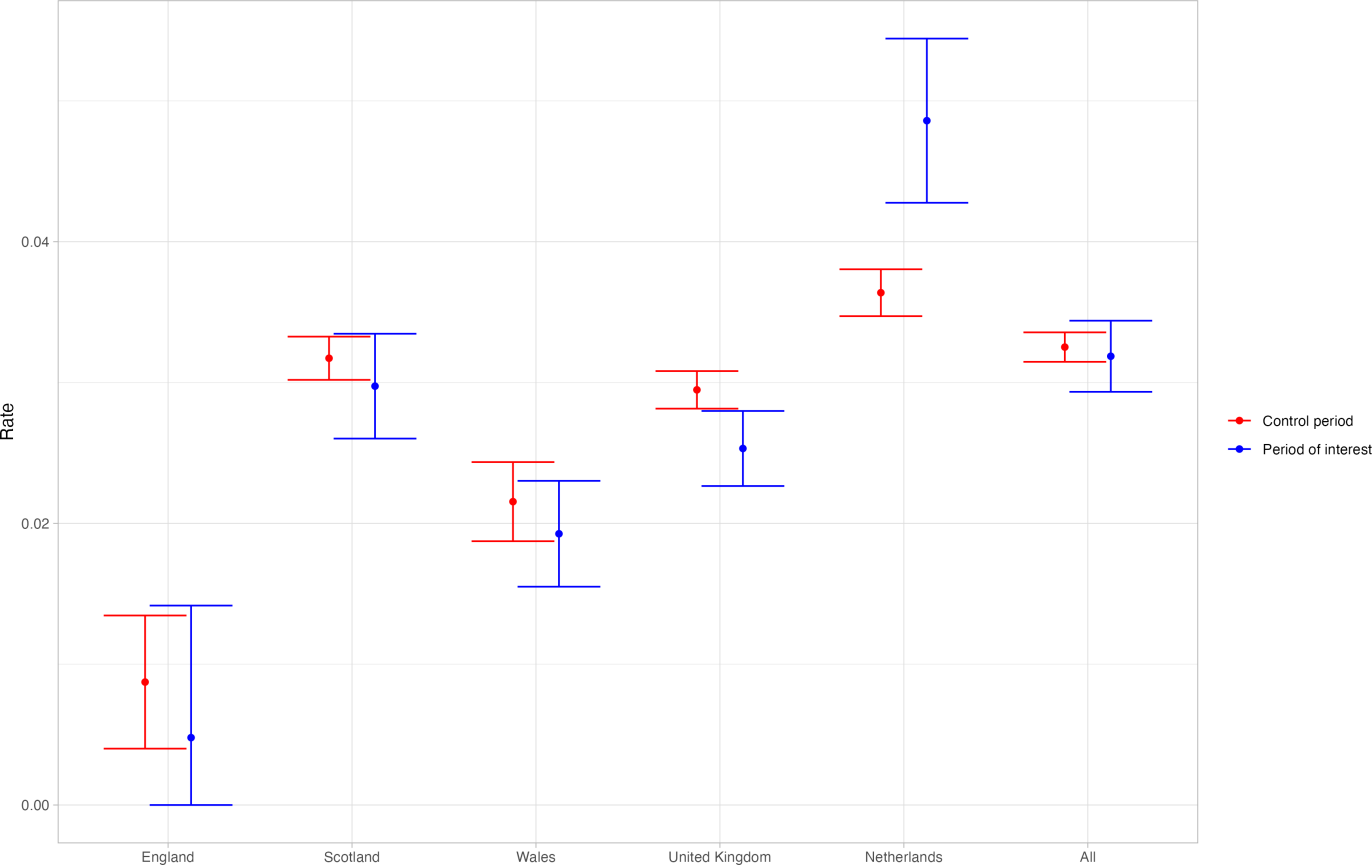
Rate of elevated (>200 U/L) ALT or AST test in 3-week- to 5-year-olds in the baseline period and the period of interest in different locations. The whiskers represent 95% CIs. ALT: alanine transaminase; AST: aspartate transaminase.

The difference in the rate of an elevated ALT or AST test finding in the baseline period compared to the period of interest can be seen in Figure 4 for the different contributing locations. There are mostly small differences between the rate in the baseline period and the period of interest, with the exception of the Netherlands, which has a relatively large increase in the period of interest compared to the baseline period.

**Figure 5.**
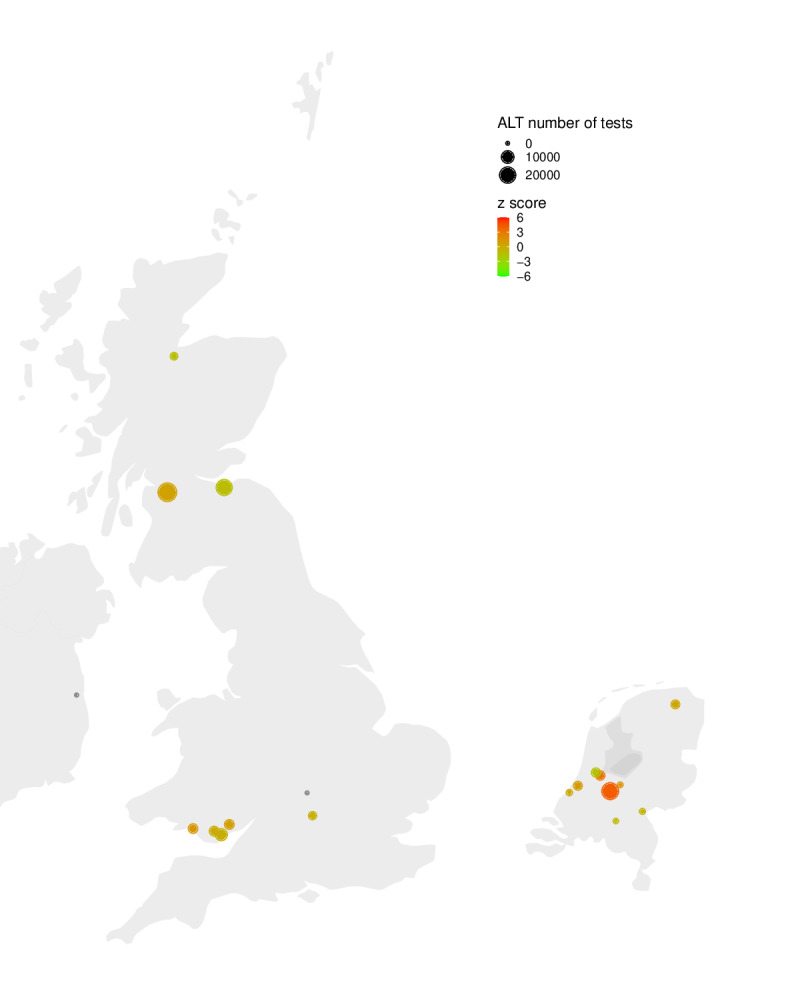
The *z* scores for the difference in the rate of elevated (>200 U/L) ALT in the period of interest (cases presenting with non–A-E hepatitis) and the baseline period (pre-2020), for 3-week- to 5-year-olds. ALT: alanine transaminase.

The *z* scores—the number of SDs that the rate for an elevated ALT test was higher or lower in the period of interest compared to the baseline period—were calculated for different contributing locations (Figure 5) and on a country level (Table 2).

There is large heterogeneity in the *z* scores, even in geographically close locations (Figure 5 and Figure S6 in Multimedia Appendix 1). For some locations, no *z* score could be calculated, because the number of tests in the baseline period (pre-2020) was too small.

**Table 2.** *z* scores for the difference in SD for the rate of elevated AST^a^ or ALT^b^ in the period of interest (cases presenting with non–A-E hepatitis) and the baseline period (pre-2020), for 3-week- to 5-year-olds, and the corresponding *P* values (United Kingdom consists of the results of England, Scotland, and Wales combined).

Location	*z* score	*P* value
England	−0.57	.57
Scotland	−0.94	.35
Wales	−0.92	.36
United Kingdom	−2.58	.01
The Netherlands	4.48	7.3×10^−6^
All	−0.46	.64

a AST: aspartate transaminase.

b ALT: alanine transaminase.

Next, data from different locations were combined to calculate *z* scores on the country level (Table 2). In the Netherlands, the *z* score was 4.48 (*P*=7.3×10^−6^), and in the United Kingdom, the *z* score was −2.58 (*P*=.01). When combining all available data, there was no significant *z* score (−0.46; *P*=.64).

### Primary Care Data

The number of recorded liver function tests decreased following the onset of the COVID-19 pandemic. The rate of AST tests remained below pre-pandemic levels in those aged 21‐30 and 11‐20 years but recovered in other age groups (Figure S7 in Multimedia Appendix 1). Consistent with our findings in the pragmatic analysis, there was no evidence of a rise in abnormal liver function test results during the period of interest (Figure S8 in Multimedia Appendix 1). An accompanying report for these results is openly available on the opensafely website [16].

## Discussion

### Summary

Our approach of sharing a simple software tool for local use enabled rapid, federated data analytics across 30 clinical centers in 3 countries. In the Netherlands, we found an increase in elevated AST and ALT tests in 3-week- to 5-year-olds in the period of interest, possibly caused by a larger outbreak of milder cases of hepatitis, mainly in Utrecht. In all the other locations, we found no evidence of a large outbreak of milder cases of hepatitis corresponding to the outbreak period, and we did not observe any clear indication of changes in primary care activity or test results in the same period.

### Findings in Context

A study by Tan et al [17] used retrospective data from a single hospital in Oxfordshire (United Kingdom) and found an increase in the number of adults presenting with acute hepatitis of unknown etiology (diagnosed using *ICD-10* (*International Statistical Classification of Diseases, Tenth Revision*) diagnostic codes or ALT values of at least 2× the ULN) at the time of the non–A-E hepatitis outbreak in children, but found no increase in the number of HAdV infections in the same age group. Similar to our results in the United Kingdom, they found no evidence of an increase in elevated AST or ALT values in any age group. Another, questionnaire-based, study in 22 European countries using data from the first months of 2022 and comparing it to previous years, found no evidence for an increase in the occurrence of severe hepatitis in children [18].

A third study reported results that differ from our results. In England, the number of emergency department visits and hospital admissions was higher in 2022 compared to the years before for children (age 1‐4 years) who presented with jaundice and liver-related (non A-E) hepatitis illness. However, there were no data available from before the COVID-19 pandemic, and health-seeking behavior may have changed during the pandemic [15].

We found differences in the rate of elevated AST and ALT tests in 3-week- to 5-year-olds in geographically close locations. Similarly, an earlier study by van Beek et al [19] showed a difference in the reported increase in cases of severe non–A-E hepatitis in countries that are geographically close. The United Kingdom, Italy, Spain, Sweden, Ukraine, and Israel reported an increase in the number of probable cases in the first months of 2022 compared to the preceding 5 years. No such increase was reported in Germany, the Netherlands, or Denmark [19]. It is possible that more granular data on the location of the cases would show a different relationship between the rate of elevated AST and ALT measurements during the period in which cases were recorded, but due to Control of Patient Information regulations, it was not possible to gather these data. Moreover, patients, especially children, may not always be admitted to the hospital nearest to them, but rather to a hospital that can provide specialized care, which also makes aggregation of the contributing locations more challenging.

Interestingly, van Beek et al [19] found no increase in cases of severe non–A-E hepatitis in the Netherlands and did find an increase in cases of severe non–A-E hepatitis in the United Kingdom. There are several possible explanations for the difference in our results. First of all, they had data from 1 hospital in the Netherlands and 5 hospitals in the United Kingdom, while our study included data from 9 Dutch hospitals and 9 hospitals in the United Kingdom. The higher number of different locations increases the possibility of picking up a local signal. Second, their study was published early in the outbreak and included data up to April 18, 2022, when cases were still being reported. In our study, the data extraction period ranged from March 2022 to December 2022. Finally, their cases involve patients between 0 and 16 years of age, while our main analysis is focused on 3-week- to 5-year-old children.

### Limitations

This approach also comes with limitations. First, AST and ALT are used as a proxy for hepatitis. In times when the causative agent is incompletely understood, syndromic surveillance for a proxy is used, but when the cause is determined, surveillance of more specific test results is typically more useful. However, surveillance for AAV2 would be complicated given that it is not a routine laboratory test. Second, there are causes other than AAV2 or HAdV infection that could contribute to an increase in the proportion of elevated AST and ALT tests. Lockdowns and a sharp decrease in social interactions delayed exposure to many respiratory viruses in young children, and since many respiratory viral infections can lead to an increase in transaminases [20,21], the increase could also be caused by an increase in influenza virus, respiratory syncytial virus or Epstein-Barr virus infections. Using our data, it is not possible to differentiate between different causes of an increase in AST and ALT, although given the increase in HAdV in stool samples found in several countries, this is a plausible explanation [22]. Moreover, peaks in positive respiratory syncytial virus or influenza virus tests in young children were only reported during a short period of the non–A-E hepatitis outbreak, and not during the entire period [23]. Third, our results only apply to the population of hospitalized children, which is likely to be different from the general population of 3-week- to 5-year-olds. Fourth, we decided to only include measurements from individuals without previously elevated ALT or AST measurements, which is a strict inclusion criterion. However, the number of children with multiple elevated ALT or AST measurements is likely to be limited, and therefore, unlikely to influence our results or conclusion. Finally, the cutoff used in our study was 200 U/L, reflecting a mild to moderate elevation, but a more subtle increase in AST and ALT elevations could possibly have been picked up with a lower threshold but comes with a higher risk of false positive results.

### Policy Implications and Interpretation

We demonstrated that it is feasible to use anonymous, aggregated, routinely collected clinical laboratory data for syndromic disease surveillance, and this approach could be used for new outbreaks of diseases with unknown etiology, widespread poisoning incidents, or if there are few or no diagnostic tests available. Hospital laboratories collect large amounts of data on a daily basis that can potentially be of use for disease surveillance, but these are currently not optimally used. Completely anonymous data can be shared lawfully and safely between institutions, without the need for extensive contracting or additional information governance; although organizations must still have an appropriate legal basis, and the United Kingdom additionally meets the common law duty of confidence, to process the routinely collected identifiable data into an anonymous ready format. Collecting summary data from different locations is an efficient way to aggregate large amounts of data and identify changes over time and in different geographical locations. Additionally, the emergence of large electronic databases containing routinely collected health data has made syndromic surveillance of communicable diseases significantly easier [24]. Moreover, because the data are routinely collected, there is little to no administrative burden for staff, which is especially relevant during outbreaks of infectious diseases. Finally, the process of collecting and aggregating data for the contributing locations was straightforward using the script, and this approach would work for many laboratory measurements. After composing the analysis script, updating the results by rerunning the code took a matter of minutes. If studies such as this one can demonstrate clinical use in monitoring rates of abnormalities then this may indicate a role for these rates to be monitored prospectively either locally or nationally.

### Conclusions

We demonstrated that the rapid collection of large amounts of summary data from different hospitals in different countries for a pooled analysis is feasible for the purpose of syndromic surveillance. In most regions, there was no evidence of an outbreak of milder cases of hepatitis during the period of interest.

### Information Governance

For the OpenSAFELY analysis, first, NHS England is the data controller of the NHS England OpenSAFELY COVID-19 Service; TPP is the data processor; all study authors using OpenSAFELY have the approval of NHS England [25]. This implementation of OpenSAFELY is hosted within the TPP environment which is accredited to the ISO 27001 information security standard and is NHS IG Toolkit compliant [26].

Second, patient data have been pseudonymized for analysis and linkage using industry standard cryptographic hashing techniques; all pseudonymized datasets transmitted for linkage onto OpenSAFELY are encrypted; access to the NHS England OpenSAFELY COVID-19 service is via a virtual private network connection; the researchers hold contracts with NHS England and only access the platform to initiate database queries and statistical models; all database activity is logged; only aggregate statistical outputs leave the platform environment following best practice for anonymization of results such as statistical disclosure control for low cell counts [27].

Third, the service adheres to the obligations of the UK General Data Protection Regulation and the Data Protection Act 2018. The service previously operated under notices initially issued in February 2020 by the the Secretary of State under Regulation 3(4) of the Health Service (Control of Patient Information) Regulations 2002, which required organizations to process confidential patient information for COVID-19 purposes; this set aside the requirement for patient consent [28]. As of July 1, 2023, the Secretary of State has requested that NHS England continue to operate the Service under the COVID-19 Directions 2020 [29]. In some cases of data sharing, the common law duty of confidence is met using, for example, patient consent or support from the Health Research Authority Confidentiality Advisory Group [30].

Fourth, taken together, these provide the legal bases to link patient datasets using the service. General practitioner practices, which provide access to the primary care data, are required to share relevant health information to support the public health response to the pandemic, and have been informed of how the service operates.

## Supplementary material

10.2196/55376 Multimedia Appendix 1 Additional methods and results.

## Abbreviations

AAV2: adeno-associated virus 2
ALT: alanine transaminase
AST: aspartate transaminase
HAdV: adenovirus
NHS: National Health Service
RIVM: National Institute for Public Health and the Environment
ULN: upper limit of normal
WHO: World Health Organization
