# Supplementary material for: Using Laboratory Test Results for Surveillance During a New Outbreak of Acute Hepatitis in 3-Week- to 5-Year-Old Children in the United Kingdom, the Netherlands, Ireland, and Curaçao: Observational Cohort Study
Source: JMIR Public Health Surveill. 2024 Dec 23;10:e55376. doi: 10.2196/55376 (PMC11770233; doi:10.2196/55376)
Supplement: Multimedia Appendix 1 [file publichealth-v10-e55376-s001.docx]

## Methods

### Health boards

The Aneurin Bevan University Health Board (ABU) consists of the Grange University Hospital, the Royal Gwent, Nevil Hall Hospital and Ysbyty Ystrad Fawr. The Cardiff and Vale University Health Board (CVU) covers the University Hospital of Wales, Llandough Hospital, and Velindre Cancer Centre. The Cwm Taf Morgannwg University Health Board (CTM) consists of the Royal Glamorgan Hospital, Prince Charles Hospital and Tonteg Hospital. Finally, Swansea Bay University Health Board (SBU) includes Morriston Hospital, Princess of Wales Hospital, Singleton Hospital and Neath Port Talbot Hospital. The location of the hospital that was most central for every health board was used for the location.

### Legal basis for sharing summary data

Hospitals were asked to share anonymous summary data by making it available to us under a creative commons (CC) license https://creativecommons.org. An Attribution 4.0 International (CC BY 4.0) license means that the creator needs to be acknowledged and the data can be used and built-upon, which allowed us to efficiently collect and summarise data from many contributors.

## Results

##

*Figure S1: Number of tests performed over time in 3-week to 5-year-olds, in (a) the UK, (b) Netherlands, and (c) all locations combined*

## AST

We examined rates for increased AST values for 3-week to 5-year-olds by location (Figure S2). No increase in rate in our period of interest was found in the Netherlands or the UK (Figure S2 (a) and Figure S2 (b)). Similar to ALT, there is a general increase in the rate of AST in the UK in 2018 (Figure S2 (a)) and in the Netherlands in 2016 (Figure S2 (b)). When pooling all available data, the increased rate can be seen from 2016 onwards compared to pre-2016 (Figure S2 (c)).

*Figure S2: Rates for elevated (>200 U/L) AST tests in the 3-week to 5-year-olds in the UK, the Netherlands and all. Shaded area represents the 95% confidence interval. The dotted line represents the first month in which cases of non A-E hepatitis were reported.*

### All age groups

There is a slight increase in the rate for elevated AST values in 3-week to 5-year-olds when pooling across contributing locations at the start of 2018 (Figure S4). A stable rate over time for elevated AST values can be seen in the older age groups, while variation is very high for the <3-week olds. A similar pattern is seen for ALT (Figure S3).

| 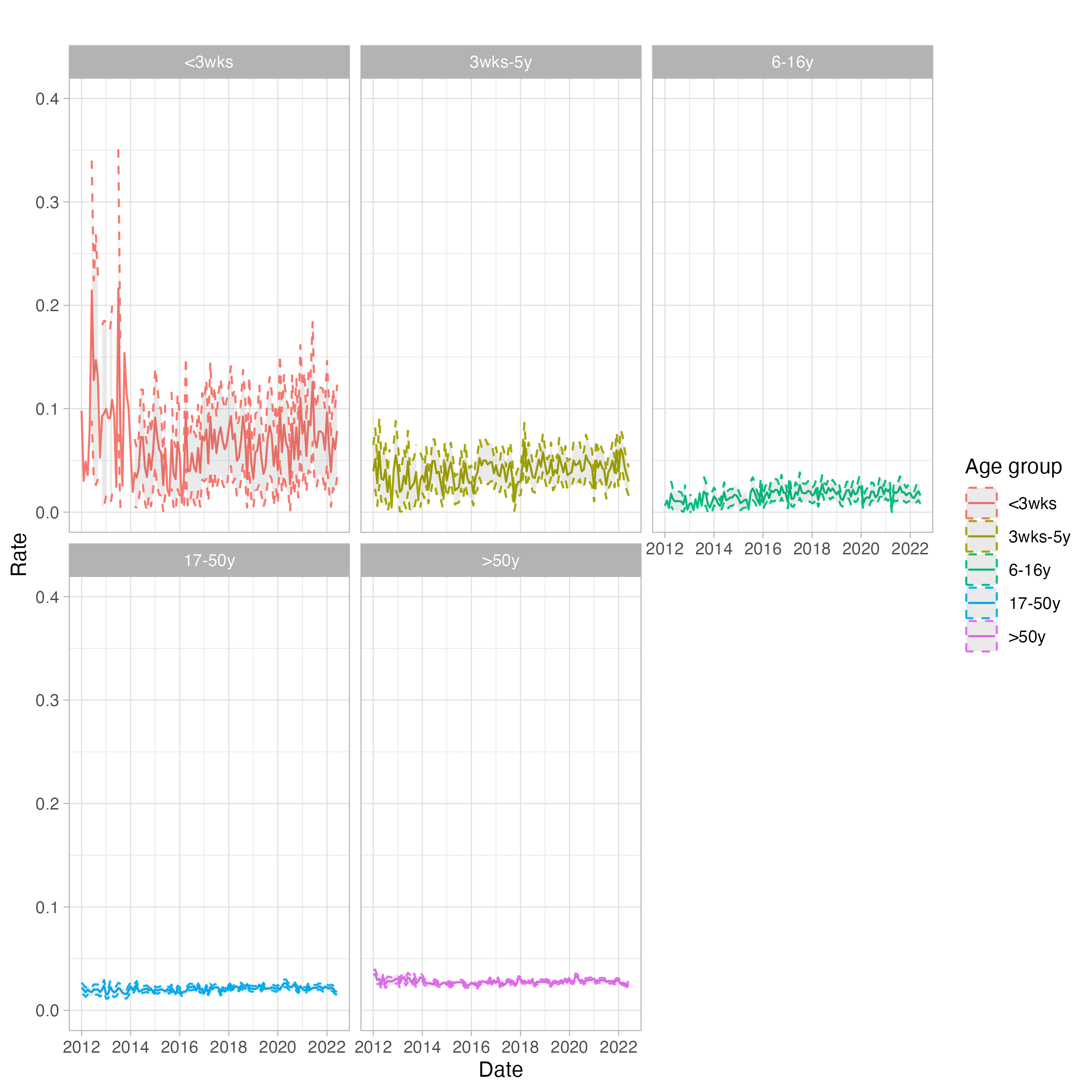  Figure S3: Rates and 95% confidence intervals for elevated (>200 U/L) ALT tests across all contributing sites |
| --- |

| 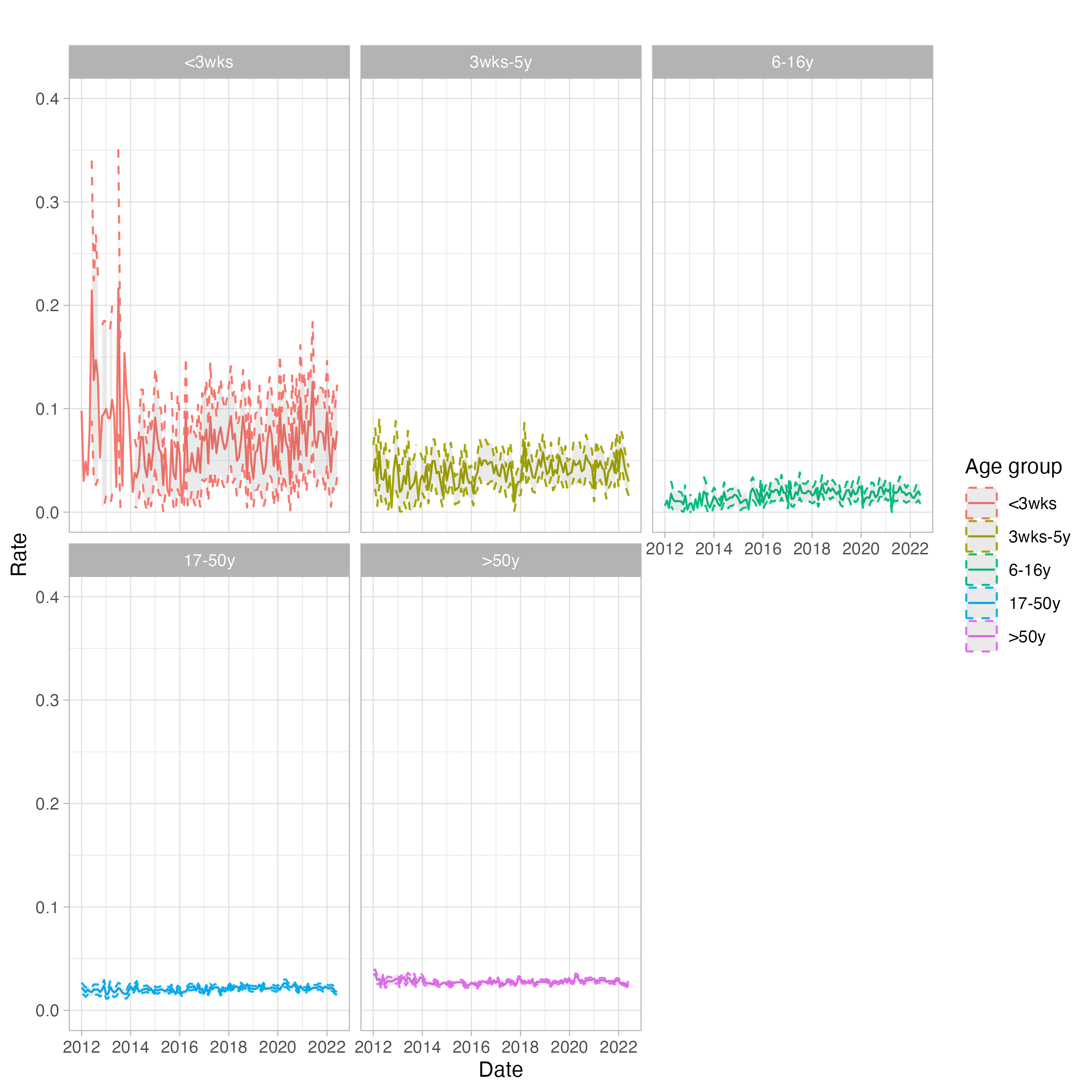  Figure S4: Rates and 95% confidence intervals for elevated (>200 U/L) AST tests across all contributing sites |
| --- |

### Elevated means

For AST, both in the UK (Figure S5 (a)) and the Netherlands (Figure S5 (b)) small increases can be seen in the beginning of 2022, but similar peaks are found several times in the years prior. A similar pattern is seen when all data is combined (Figure S5 (c)).

*Figure S5: Mean AST value (U/L) in the 3-week to 5-year-olds with elevated (>200 U/L) ALT measurements in (a) the UK, (b) the Netherlands and (c) all. Shaded area represents the 95% confidence interval. The dotted line represents the first month in which cases of non A-E hepatitis were reported.*

| 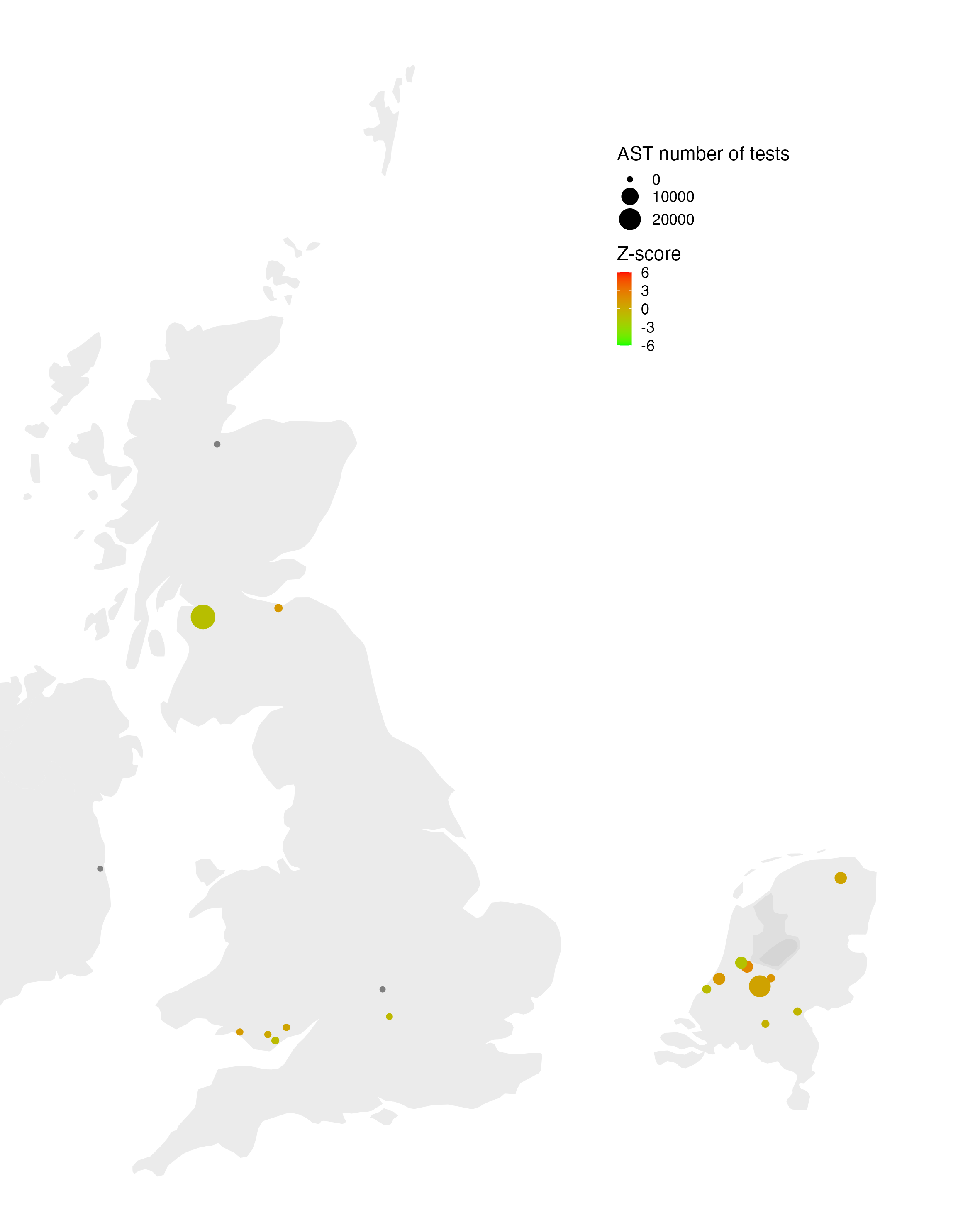  Figure S6: Z-scores for the difference in SD for the rate of elevated (>200 U/L) AST in the period of interest (cases presenting with non A-E hepatitis) and the control period (pre-2020), for 3-week to 5-year-olds. |
| --- |

## Primary care data

| 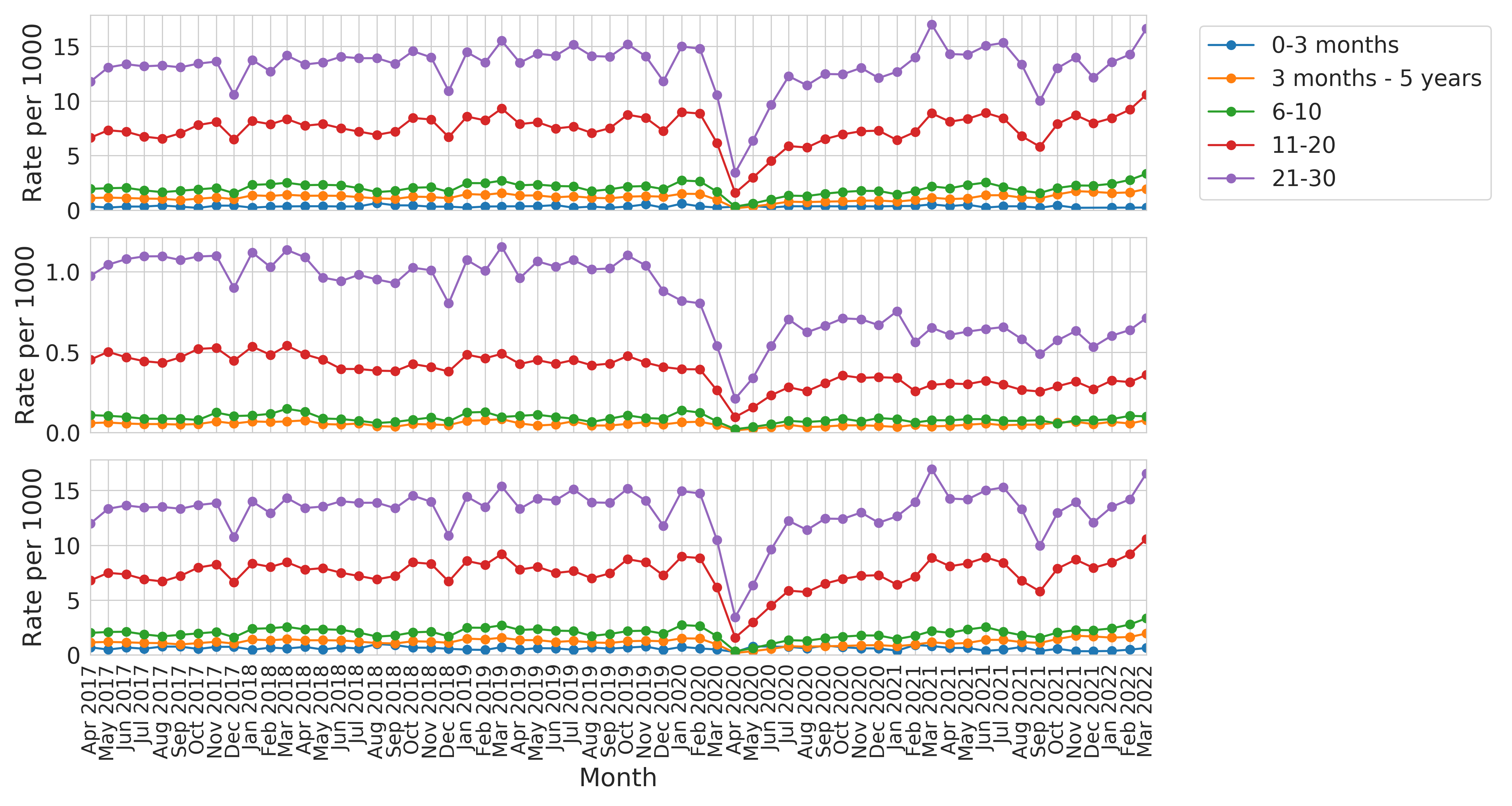  *Figure S7: Monthly rate per 1000 patients of recorded codes relating to (A) ALT tests, (B) AST tests and (C) bilirubin tests between April 2017 and March 2022 by age group. Rates where the underlying count is <=7 have been redacted.* |
| --- |

| 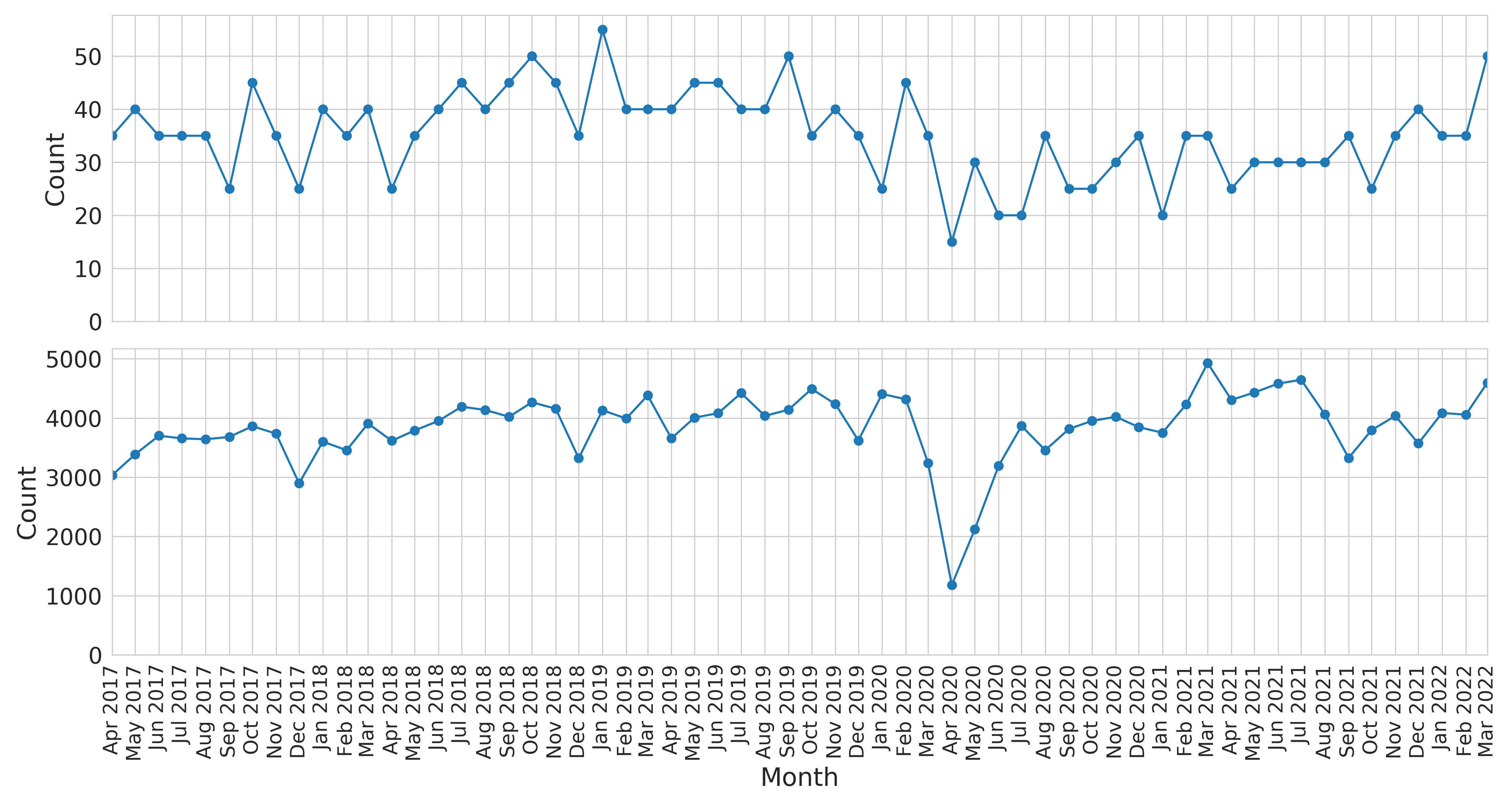  *Figure S8: Count of (A) ALT tests and (B) bilirubin tests out of range between April 2017 and March 2022 in patients aged <=30 years. All counts are rounded to the nearest 5. Monthly counts for AST tests were <=7 and are therefore not shown.* |
| --- |

### Sensitivity analysis

As a sensitivity analysis, data provided by Curaçao was included in the analysis of all locations combined. The rate for elevated (>200 U/L) measurements over time for the different age groups, can be found in Figure S9 (ALT) and Figure S10)(AST). Elevated means for the 3-week to 5-year olds can be seen in Figure S11. Since there was relatively little data from Curaçao, the results are almost identical. The Z-score including data from Curaçao to data from all other locations was also the same as the results excluding data from Curaçao (Z-score 0.64, P-value 0.64).

| 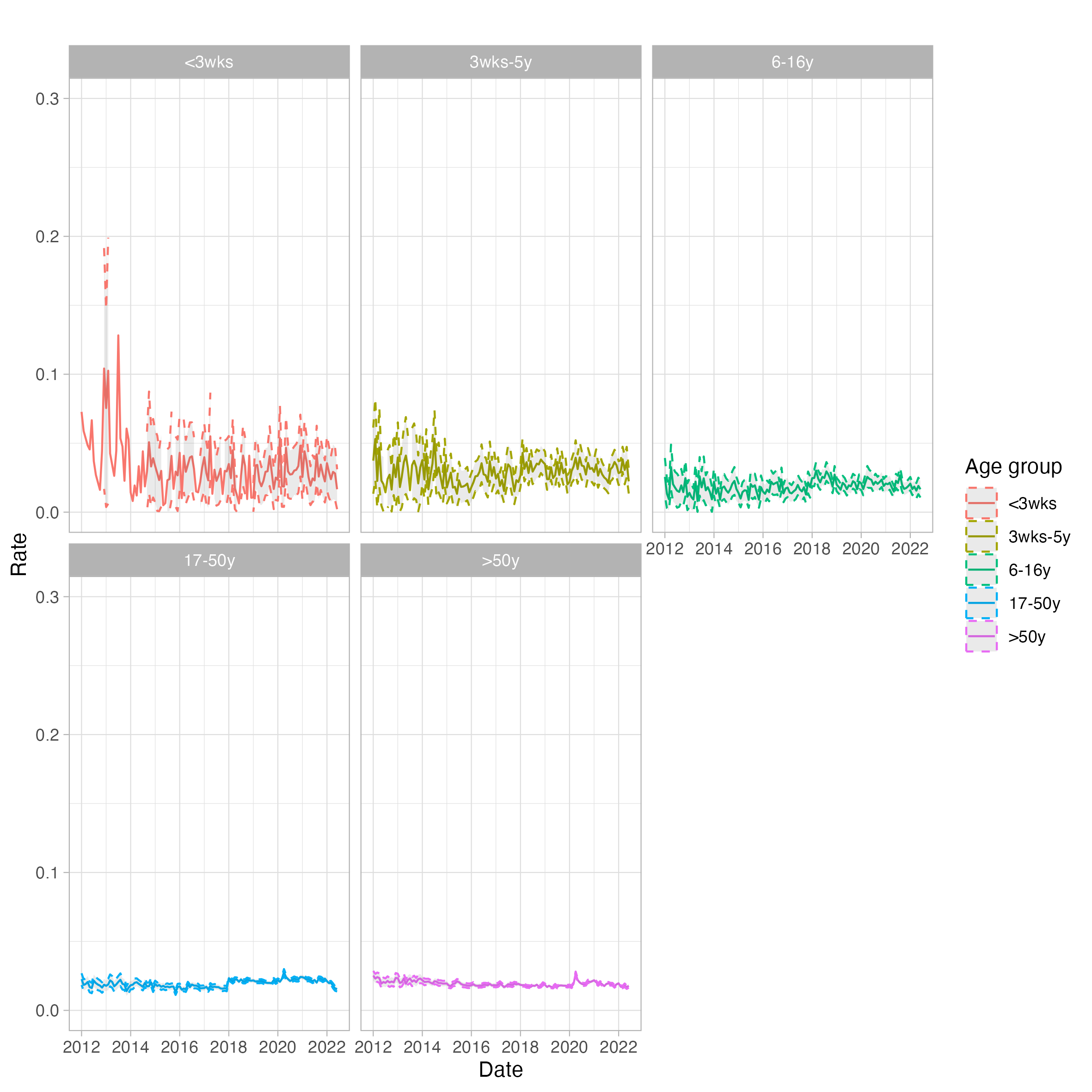  Figure S9: Rates for elevated (>200 U/L) ALT when adding data from Curaçao to all other locations. Shaded area represents the 95% confidence interval. |
| --- |

| 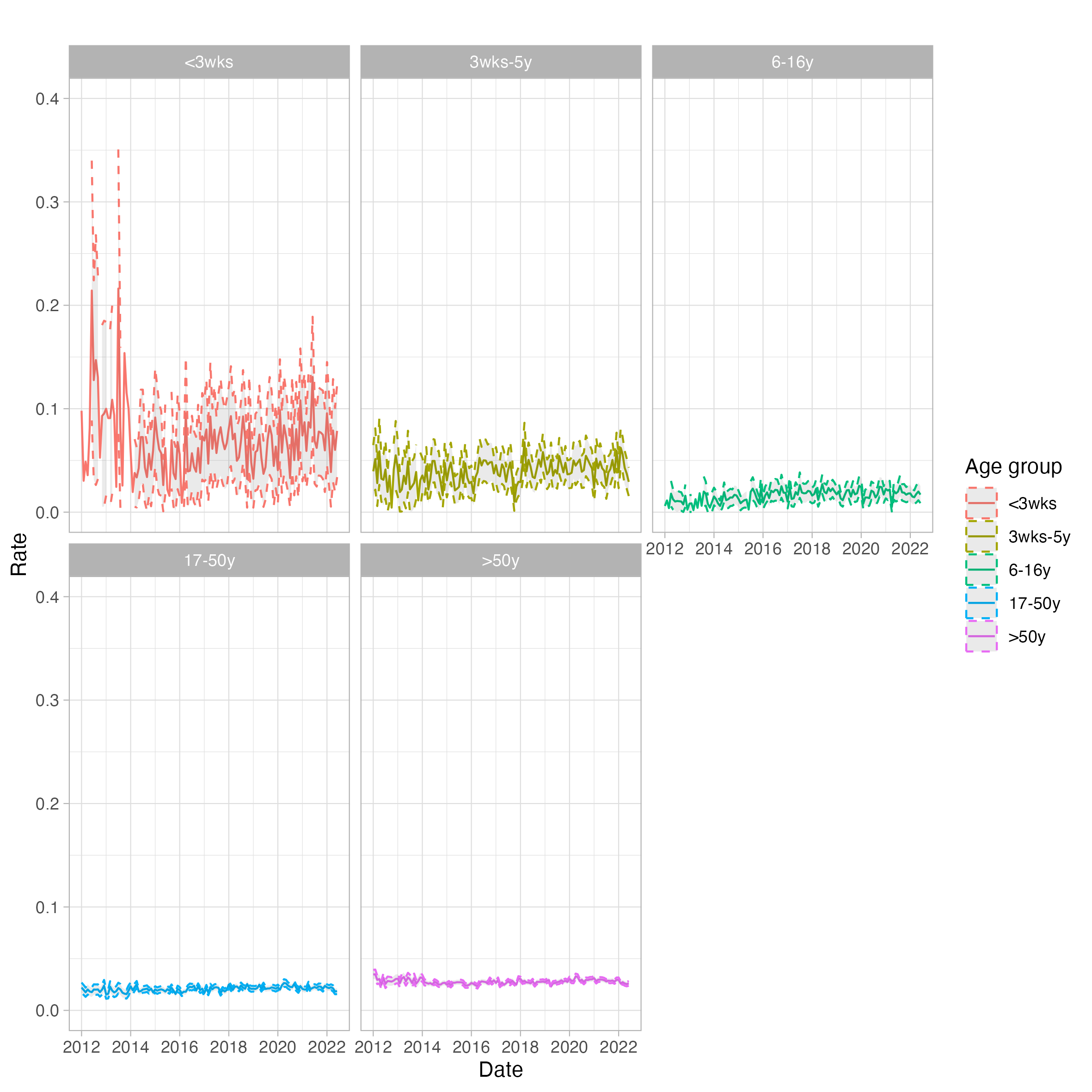  Figure S10: Rates for elevated (>200 U/L) AST when adding data from Curaçao to all other locations. Shaded area represents the 95% confidence interval. |
| --- |

**

*Figure S11: Mean a) ALT and b) AST value (U/L) in the 3-week to 5-year-olds with elevated (>200 U/L) measurements when adding data from Curaçao to all other locations. Shaded area represents the 95% confidence interval. The dotted line represents the first month in which cases of non A-E hepatitis were reported.*

# Hepatitis study group

### Royal Alexandra Hospital, Paisley, UK

Iain Jones

### Raigmore Hospital, Inverness, UK

Heidi Mendoza, John Quinn, Claire Paulin, David Cameron

### Royal Infirmary Edinburgh, Edinburgh, UK

Carol Thomson

### The Francis Crick Institute, London, UK

Gavin Kelly, Philippa Matthews, Philip East

### Nuffield Department of Medicine, University of Oxford, UK

Nicole Stoesser

### Oxford University Hospitals NHS Foundation Trust, Oxford, UK

Tim James

### St. Vincent’s University Hospital, Dublin, Ireland

Janice Reeve, Patrick Twomey, Alistair Nichol

### Leiden University Medical Center, Leiden, The Netherlands

Maaike C Swets, Geert H Groeneveld, Paul W Schenk, Leo G Visser

### Meander Medical Centre, Amersfoort, The Netherlands

Merel van Wijnen, Ryan A Basdew

### Canisius-Wilhelmina Hospital, Nijmegen, The Netherlands

Paul Geutjes

### Amsterdam University Medical Centre, Amsterdam, The Netherlands

Johan C Fischer, Hans HM Schotman

### University Medical Center Groningen, Groningen, The Netherlands

Jenny E Kootstra-Ros

### Haga Hospital, The Hague, The Netherlands

Martijn Dane

### Jeroen Bosch Hospital, Den Bosch, The Netherlands

Ron Kusters

### University Medical Center Utrecht, Utrecht, The Netherlands

Hans Kemperman

### Analytisch Diagnostisch Centrum, Willemstad, Curaçao

Nasser E Ajubi

### Centre for Infectious Disease control, Institute for Public Health and Environment, Bilthoven, The Netherlands

Diederik Brandwagt, Roan Pijnacker

### Aneurin Bevan University Health Board, Wales

Jessica Harris
